# A Proving of Lager Beer

**Published:** 1884-04

**Authors:** Samuel Swan

**Affiliations:** New York


### PROVING OF "LAGER BEER," WITH CASES ILLUSTRATIVE OF ITS ACTION.

A distinguished physician in a neighboring city requested me to send him a high potency of "Lager," if I thought that with it he could cure a lady of an undue foundness for that beverage.

I sent him the mm. potency (Swan's notation), requesting him to watch carefully for any symptoms he could attribute to the action of the drug.

He gave the patient one dose June 27th, 1883. He writes me as follows: "Her symptoms after taking the drug were like a raging furnace flame in the entire urinary apparatus, with a fierce burning desire to urinate constantly, but it passed from her only in drops. These symptoms, evidently the effect of Lager, induced me (him) to think of it in the following case.

"October 27th.—Mr. ——, aged over sixty-five, complained of a burning, fiery heat in the renal region, which passed upward to the neck, head, and mouth, causing a burning, parched tongue, compelling him to keep his mouth closed, and thus breathe only through his nostrils. This heat produced a sense of constriction around his neck, and made him feel as if he could not breathe, and as if everything about his neck and chest was too tight.

"The burning, fiery flame also passed down from kidneys, through both ureters to bladder and urethra.

"He was compelled to urinate from three to six times every night, and very often during the day. The stream was hot, small, direct, and no signs of any urethral stricture, but the suffering during the passage of the water *was awful*, and at the close of each micturation, day or night, the urine dribbled annoyingly.

"Great lameness or soreness in his back in the renal region; the suffering was aggravated when he arose to walk, or a few minutes after being seated.

"Now all this had been coming on gradually for about fifteen years, but the madness of the flame came within the past few weeks, and he had thus far failed to obtain any relief from treatment.

"I asked him to sit down, to rise up, and to walk up and down the hall. Each motion horribly aggravated the suffering. I then gave him on his tongue about fifty pellets No. 6 of Lager mm. and waited about ten minutes for results, and then directed him to again try to rise and walk; to his astonishment he found he could do it with less suffering than before. I then detained him thirty minutes longer, and when he left he remarked that his back did not trouble him near as much as it did when he came.

"October 31st.—Patient again appears with a more cheerful countenance, and very thankful he was better. He now only rose once in the night, urinated with very little burning, and *no dribbling;* during the day he urinated two or three times in eight hours; the dribbling was slight and hardly noticed. There was no suffering in the back, except a little soreness and tenderness at times; still had some 'flames' rising up the back to the neck, but slight, and he cannot detect any of his former cruel sufferings.

"November 8th.—Patient called this morning to show me how well he is. He voted on Tuesday, and was full of joy; no symptoms.

"The lady patient to whom Lager was first given reports that she is entirely well, and her friends say 'She now does not drink lager at all.'

"I have given it in the case of intense heat in the hypogastric region, extending like a flame up the left side to the region of the heart with frequent urinations in small quantities; water very high colored, staining the vessel with a reddish deposit. Patient reports 'very much better in every respect, but not quite well.'

SAMUEL SWAN.

"N. B. I have since learned that the male patient, never used alcoholic drinks, nor lager more than three times in his life, and many years since left off tobacco.

"N. B. 2. Provers should know that the best results *cannot* be obtained by taking the remedy by the 'schooner.'

"With the 1m. potency they will get the characteristics."

